# California dragonfly and damselfly (Odonata) database: temporal and spatial distribution of species records collected over the past century

**DOI:** 10.3897/zookeys.482.8453

**Published:** 2015-02-16

**Authors:** Joan E. Ball-Damerow, Peter T. Oboyski, Vincent H. Resh

**Affiliations:** 1 Department of Environmental Science, Policy & Management, University of California, Berkeley, California 94720-3114, USA; 2 Essig Museum of Entomology, University of California, Berkeley, California, USA

**Keywords:** Museum specimens, observational records, bias, change in distribution, species richness, digital catalog

## Abstract

The recently completed Odonata database for California consists of specimen records from the major entomology collections of the state, large Odonata collections outside of the state, previous literature, historical and recent field surveys, and from enthusiast group observations. The database includes 32,025 total records and 19,000 unique records for 106 species of dragonflies and damselflies, with records spanning 1879–2013. Records have been geographically referenced using the point-radius method to assign coordinates and an uncertainty radius to specimen locations. In addition to describing techniques used in data acquisition, georeferencing, and quality control, we present assessments of the temporal, spatial, and taxonomic distribution of records. We use this information to identify biases in the data, and to determine changes in species prevalence, latitudinal ranges, and elevation ranges when comparing records before 1976 and after 1979. The average latitude of where records occurred increased by 78 km over these time periods. While average elevation did not change significantly, the average minimum elevation across species declined by 108 m. Odonata distribution may be generally shifting northwards as temperature warms and to lower minimum elevations in response to increased summer water availability in low-elevation agricultural regions. The unexpected decline in elevation may also be partially the result of bias in recent collections towards centers of human population, which tend to occur at lower elevations. This study emphasizes the need to address temporal, spatial, and taxonomic biases in museum and observational records in order to produce reliable conclusions from such data.

## Introduction

Natural history specimens are arguably the most valuable records of the historical occurrence of organisms. In contrast to scientific publications, which usually are most relevant for the first ten years following their appearance, information from specimens becomes more valuable with age (Winker 2004). Museum records that are backed by voucher specimens also allow researchers to verify species identification. In addition to their traditional use in taxonomy and biogeography studies, specimens can provide a wealth of information concerning changes in morphology, genetic and biochemical composition, and the distribution and diversity of organisms over time (Cao et al. 2013, Graham et al. 2004, O’Connell et al. 2004, Pyke and Ehrlich 2010, Winker 2004). However, large-scale applied and ecological studies using museum specimens are exceedingly difficult to conduct without a database of existing records. While the development of digital catalogs of natural history specimens began in 1970, by 2010 only ~ 3% of total records worldwide were estimated to be available online through the mobilization efforts of the Global Biodiversity Information Facility (GBIF 2014; Ariño 2010).

Many vertebrate collections have complete or near-complete databases of their specimens, along with ancillary information such as photos, field notes, and published manuscripts associated with particular specimens (e.g. Guralnick and Constable 2010, Pyke and Ehrlich 2010). However, databases for insects and other invertebrates have lagged far behind vertebrates (Schuh et al. 2010). This is largely because the task of databasing information from millions of small specimens, which represent the most diverse animal group on the planet, is enormous. In addition, these collections often lack the necessary resources to meet desired specimen curation because insects tend to undergo continual taxonomic revision (DeWalt et al. 2005). Therefore, many have considered digitization of huge collections of insects with tiny and highly abbreviated labels to be impossible (Schuh et al. 2010). However, in response to a growing need for specimen data in research, more insect and other large natural history collections are in the process of undergoing or beginning digitization (e.g. Abbott 2005, Favret and DeWalt 2002, Graham et al. 2004, Hill et al. 2012, Schuh et al. 2010). In the United States, the National Science Foundation (2014) has made such efforts possible through funding initiatives, including the Advancing Digitization of Biodiversity Collections (ADBC) and the Thematic Collections Network (TCN).

Along with digitization, however, comes the responsibility of database curators and data-users to acknowledge and address the many biases that exist in specimen data. Because the approach of natural history collection acquisition and management has traditionally focused on taxonomic work and the special interests of curators and enthusiasts (Graham et al. 2004), the data are usually biased in regards to the species collected and the temporal and spatial distribution of records (Pyke and Ehrlich 2010). For example, collectors have often focused collecting efforts on rare, large, and charismatic species while neglecting more common or cosmopolitan species (Winker 2004). Collections also tend to occur along roads, railroad tracks, or near centers of human population (Graham et al. 2004, Pyke and Ehrlich 2010). There is usually a strong correlation between collection effort, or number of records, and the number of species documented for a given time period or region (Fattorini 2013). Therefore, well-sampled regions may have better species representation than less-sampled areas as a result of sampling effort. Such biases present in natural history collections can be reduced by incorporating as much data as possible in occurrence-based analyses of the data. For example, compiling records from multiple institutions may help reduce the problem of localized collecting from any one collection (Pyke and Ehrlich 2010, Soberon et al. 2000).

The present study summarizes a recently completed database of Odonata records from throughout the state of California, USA, including both specimens and observational records. This group of aquatic insects provided a good starting point for a statewide database of insect specimens because they are less diverse than most insect orders, have well-known taxonomy (Clausnitzer et al. 2009), are charismatic to the general public, and have naturalist sightings that are available to supplement recent occurrence records (Abbott 2005, Odonata Central 2014). Odonata are also known to be useful indicators of freshwater ecosystem health, and are thus likely to contribute to our understanding of general response to changes in aquatic habitat and water quality (e.g. Clausnitzer 2003, Smith et al. 2007). Here, we outline the methods used in the development of the California Odonata database. We then present the spatial and temporal distribution of records to identify data gaps and biases. We determine contributions of different collection types (e.g. university and government institutions, observation-based records) to total number of records and unique county records. Finally, we assess the prevalence of records for each Odonata species before 1976 and after 1979 to determine both potential taxonomic biases and changes in species prevalence, altitude, and elevation ranges over time. We chose the time periods of before 1976 and after 1979 because they have approximately equal numbers of records, and the time period beginning in 1980 marks the beginning of accelerated temperature warming.

## Methods

### Odonata specimen database

We developed a database of Odonata occurrence records in conjunction with a larger project, known as Calbug, whose goal is to database over one million California arthropod specimens (Calbug 2014). Calbug is a collaborative project among the ten major entomology collections in California, including: the California Academy of Sciences (CASENT), California State Collection of Arthropods (CSCA), Los Angeles County Museum (LACM), San Diego Natural History Museum (SDNHM), Santa Barbara Museum of Natural History (SBMNH), Essig Museum of Entomology of the University of California at Berkeley (EMEC), Bohart Museum of Entomology of the University of California at Davis (UCBME), Entomology Research Museum of the University of California at Riverside (UCRCENT), Museum of Natural History of the University of California at Santa Cruz (UCSC), and the Oakland Museum of California (OMC). The Odonata database includes records from CASENT, CSCA, LACM, EMEC, UCBME, SBMNH, SDNHM, UCRCENT, and OMC.

In addition to the Calbug institutions, we obtained specimen data from the two largest Odonata collections in the United States, the Museum of Zoology at the University of Michigan (UMMZI) and the Florida State Collection of Arthropods (FSCA), which includes records from International Odonata Research Institute (IORI), Louisiana State Arthropod Collection (LSUC), and the Museum of Zoology Pontifical Catholic University of Ecuador (QCAZ) collections. We then incorporated data from other online databases that contain California odonate material, including that of the Illinois Natural History Survey (INHS 2014), and the National Museum of Natural History (NMNH 2014). We also included California odonate occurrence records from the personal collections of D.R. Paulson (DRPC), R.W. Garrison (RWGC), S.D. Gaimari (SDGC), and the author (J.E.B-D, Ball-Damerow et al. 2014). Finally, the odonate records of C.H. Kennedy (1917), collected throughout central California in 1914–15 are incorporated as a private collection. These records are included in the Essig museum’s online specimen database (Table 1, Essig Museum of Entomology Collections Specimen Database 2014).

**Table 1.** All contributing data sources, abbreviations, and total number of specimens.

Source collection	Abbreviation	# Specimens
CalBug Institutions		14,207
California Academy of Science	CASENT	2,876
UC Riverside	CIS	531
California State Collection of Arthopods	CSCA	24
Essig Museum	EMEC	5,550
LA County Museum	LACMENT	2,032
Oakland Museum	OMC	107
Santa Barbara Museum of Natural History	SBMNHENT	153
San Diego Natural History Museum	SDNHM	88
UC Bohart Museum	UCBME	2,776
UC Riverside	UCRCENT	70
non-CalBug Institutions		5,803
Florida State Collection of Arthropods	FSCA	65
International Odonata Research Institute (at FSCA)	IORI	3,230
Louisiana State University	LSUC	48
Museum of Zoology - Pontifical Catholic University of Ecuador (P.U.C.E)	QCAZ	12
Illinois Natural History Survey	INHS	96
University of Michigan Museum	UMMZI	1,425
US National Museum	USNM	927
Personal		3,746
C.H. Kennedy	CHK	1,190
D.R. Paulson	DRPC	930
R.W. Garrison	RWGC	576
S.D. Gaimari	SDGC	132
J.E. Ball-Damerow field collections	JEBD	918
Observations		8,269
Cal Odes	Cal Odes	6,777
Odonata Central	Odonata Central	1,492
**Grand Total**		**32,025**

Odonata was a high priority group for the Calbug project, which began in 2010. At the start of the project, we directly entered data from specimen labels into the Essig database, and assigned each specimen a Unique Identifier (UID) that is associated with the physical specimen and its database record. The Essig database uses Linux, Apache HTTP Server, MySQL, and Perl/PHP (LAMP) technology, and currently contains 117 fields based on Darwin Core standards. A Darwin Core-Archive is created monthly and made available to GBIF and other aggregators via the Berkeley Natural History Museums (BNHM) IPT service.

Since 2011, we have photographed specimens with their collection labels as the first stage of the data collection process. Further details on the imaging process are described on the Calbug website (2014). The images are then uploaded into the Essig database with species name and UID information, and stored in the database as part of the specimen record. Individuals may then enter label information for specimen records online through the Essig database, using the magnified specimen image.

### Observation-based records

In addition to specimen collections, we also included occurrence data from Odonata Central and CalOdes enthusiast observations, of which records have often been photo-vouchered and verified by odonate experts. Odonata Central (2014) is a North American database with georeferenced records, and includes photo-vouchered sightings, records from literature, and some specimen-based data (Abbott 2005). CalOdes is a California statewide dragonfly enthusiast group composed of around 125 members who track and submit lists of species observed at specific locations and dates (Dragonflies of California 2014).

### Data quality

To facilitate quality control during data entry, the Essig database uses controlled vocabularies, such as dropdown lists, date range validation, and species name authority files to validate names. Hierarchical information is automatically filled in for geography and taxonomy.

Following data entry, we conducted a data checking procedure to minimize likely data-entry errors. This included an assessment of records with the same localities for spelling errors and to determine whether locations were associated with the correct county in the state. The data entry form of the database automatically filled information from one record to the next so that records with the same information in a series did not have to be entered multiple times. To minimize carry-over errors, we therefore checked records with adjacent UIDs for questionable repeated fields, such as collector or date. Finally, we spot checked all fields for a portion of specimens against the specimen label photograph.

Odonata have been relatively well-curated in these collections over time, so that correct specimen identification was assumed in most cases. An Odonata specialist, T. Manolis (2003), recently checked most taxonomic identifications of Odonata specimens from the Calbug institutions. Odonata specimens at UMMZI and FSCA have also been curated by odonate specialists, including L.K. Gloyd and M.F. O’Brien at UMMZI, and W.F. Mauffray at FSCA.

We compared all specimen records to current county records and known distribution ranges as a method to check for outliers. Each specimen that fell outside of current county records for the species was checked for accurate identification and potential data entry errors. From these records, we retained only those with verified species identification and locality information. Finally, we corrected any species with outdated names, based on taxonomic classifications in Odonata Central (2014).

### Georeferencing

We georeferenced occurrence localities using the standardized point-radius method (Wieczorek et al. 2004). This method outlines a series of rules to assign geographic coordinates to text descriptions of locations. Using this standard, we also assigned an uncertainty estimate (i.e. radius) based on common sources of uncertainty, such as the extent of a named place (e.g. Berkeley, California) and the distance precision provided for an offset direction (e.g. 4 miles north of Berkeley, California, which has a distance precision of 1 mile). In most cases, we used multiple online georeferencing tools, including Geolocate (Rios and Bart 2010), Georeferencing Calculator (Wieczorek et al. 2004), ACME Mapper (2014), Geographic Names Information System (GNIS; 2014), and Earth Point (2014).

After all records were georeferenced, we spot checked a portion of records for accuracy. In addition, we checked all localities with listed counties that did not match county polygons using ArcGIS Desktop, release 10.1 (ESRI 2012). We then corrected any aberrant records or further investigated related records, as needed.

### Taxonomic, temporal and spatial summary of records

We first summarized the number of species within each of the families found in the state. To demonstrate the temporal and spatial coverage of species occurrence records, we then summarized records by decade, by county, and in maps of occurrence locations. For this and all subsequent analyses, we removed any species considered to be vagrant, with only one sighting in the state. We determined species richness and the total number of specimens before 1900 and by decade in the following years. We then calculated species richness and total number of records by county for the entire period of record. In order to assess the effect of effort on species richness by county, we plotted the total number of species against the number of records for each county. We also used this information to identify regions that are currently underrepresented in the collections. Finally, we mapped all Odonata occurrence locations before 1976 and after 1979 to illustrate the spatial distribution of records for these time periods.

### Contribution of collection types to county records

The four collection types included in the database were the Calbug institutions (California University and government collections), non-Calbug (non-California) institutions, private collections of odonate specialists, and observation-based records. We first summarized the total number of records from each data source. To illustrate how different collections have contributed to our knowledge of spatial distribution of odonates in the state, we determined the number of unique county records from each of the major collection types. We summarized the number of unique county records (by species and county) shared by one, two, three, or all four types.

### Species occurrence records

The final goal of this paper was to assess the prevalence of records for individual Odonata species before 1976 and after 1979 to determine both potential taxonomic biases and changes in species prevalence, altitude, and elevation ranges over time. We chose these time periods because they have comparable numbers of unique-species occurrence records (8,431 before 1976 and 9,156 after 1979). The four year gap, including the years of 1976–1979, separates the two time periods for temporal comparison while maximizing our ability to achieve similar numbers of records. Moreover, temperature began increasing rapidly starting around 1980 as a result of climate change (IPCC 2013). We removed all species that were recorded in fewer than two instances because these were considered to be vagrant species. We then determined the first and last year of documented occurrence, and the total number of records before 1976 and after 1979. We considered the total number of unique records for each time period to be a proxy for collection effort. To account for differences in collection effort, we divided the number of unique occurrences of each species by the total number of unique occurrences across all species for the respective time period. We then identified species with changes in occurrence records that are likely to result from taxonomic biases, and those that may have legitimately increased or declined in prevalence. Related studies by Ball-Damerow et al. (2014) and Manolis (2003), and expert opinion were applied to distinguish between species with actual change in prevalence over time and species with change likely resulting from taxonomic collection biases.

To determine whether species have expanded to higher latitudes or elevations, we calculated the average and range of latitude and elevation for each species before 1976 and after 1979. Any records with greater than 4 km error radius were removed from this analysis. Wilcoxon signed-rank tests were performed to determine whether the median difference in latitude and elevation means between the two time periods were significantly different.

## Results

### Database summary

There were 32,025 records from all combined sources (Suppl. material 1, Table 2). The majority of records (21,648) came from Calbug efforts. CalOdes, Odonata Central, recent field collections (Ball-Damerow et al. 2014), and C.H. Kennedy’s collections (Kennedy 1917) contributed 6777, 1492, 2016, and 1190 records, respectively (Table 2). Many of these records were not unique, and the summed total number of unique species, year, and locality combinations for all data sources was 19,000, and the total species, year, and county combinations was 13,255 (Table 2).

**Table 2.** Summary of total California Odonata records, and unique species records by year and either locality or county. Specimen database includes Calbug Institutions (California University and government-based collections), non-Calbug institutions, and private collections.

Data source	Total records	Unique locality records	Unique county records
Specimen database	21,648	11,149	8,716
C.H. Kennedy (1917)	1,190	527	404
J.E. B-D field collections	918	856	514
CalOdes	6,777	5,463	2,698
Odonata Central	1,492	1,005	923
Totals	32,025	19,000	13,255

### Taxonomic, temporal and spatial summary of records

There are currently 106 species within nine families that are known to occur in the state, including nine species of Aeshnidae, two species of Calopterygidae, 30 species of Coenagrionidae, one species of Cordulegastridae, six species of Corduliidae, 12 species of Gomphidae, seven species of Lestidae, 38 species of Libellulidae, and one species of Petaluridae. The earliest records in the database were from 1879, and include two specimens of *Argia vivida* Hagen from the Santa Ana River in Southern California, and several records of *Hetaerina americana* (Fabricius) and *Libellula saturata* Uhler in Colton, San Bernardino County, California. These specimens are all held at INHS. The last year of record in the database was 2013.

**Figure 1.**
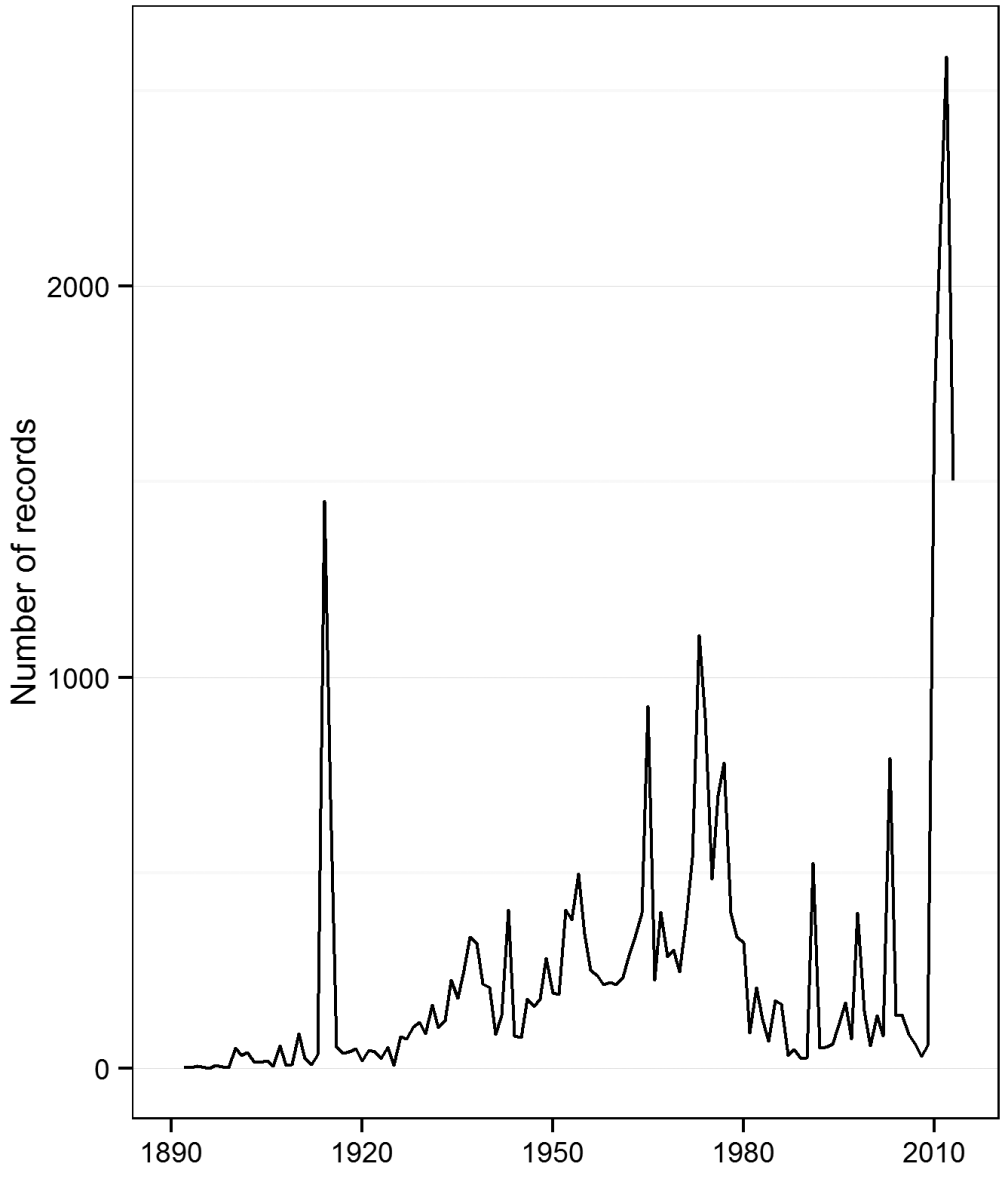
Total number of California Odonata records per year.

The first peak in Odonata collections in California occurred in 1914–1915 with C.H. Kennedy’s collections throughout the state (Kennedy 1917, Fig. 1). Subsequent peaks occurred in the mid-1950s, 1960s, and 1970s, with the largest collections from D. Paulson, R. Garrison, and S. Dunkle (Fig. 1). Most of the recent records come from CalOdes sightings and field surveys by J.E.Ball-Damerow over the period of 2010–2013.

**Figure 2.**
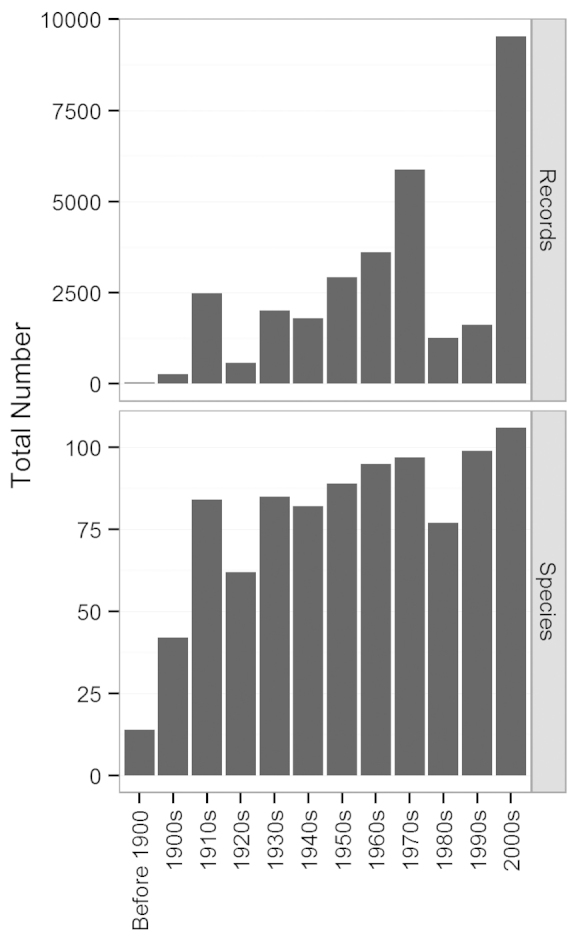
Total number of records and number of species by decade.

The total number of species found throughout the state varied only slightly by decade, except for time periods when there were less than ~ 1,200 total records, e.g. before 1900 and 1900–1910. The time period with the highest number of records and species was 2000–2013, with 9,535 records and 106 species, followed by the 1990s, with 99 species and only 1,623 total records (Fig. 2). The 1910s, which include C.H. Kennedy’s surveys, contribute 2,485 total records for 84 species (Fig. 2).

**Figure 3.**
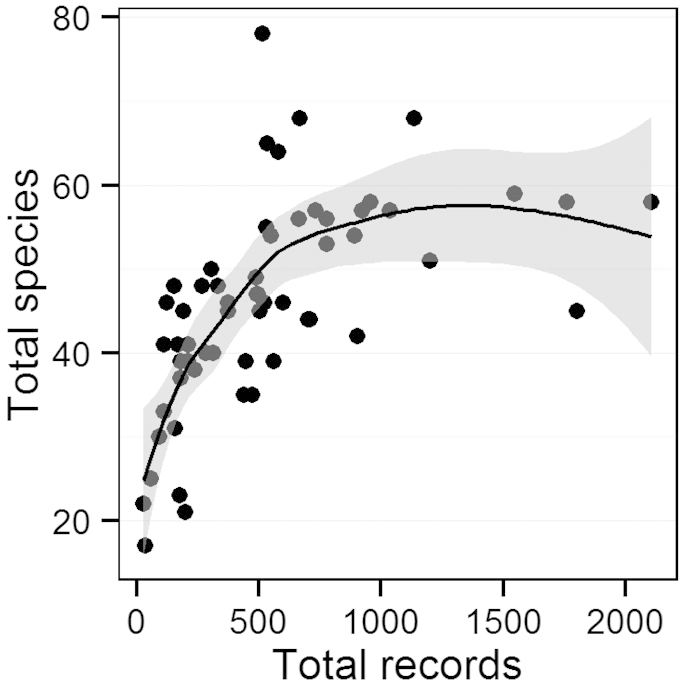
Relationship between species richness and total number of records by county, where each point represents a California county.

There was an exponential relationship between the total number of unique records from a given county and species richness observed (Fig. 3). The richness increased dramatically through ~ 600 total records, leveling off at ~ 58 species. Therefore, many counties with less than 600 records are likely to show higher species richness with increased sampling. The least-sampled county was Kings County, with only 28 records and 22 total species (Table 3). Riverside County was the most sampled with 2,108 unique records and 58 species observed (Table 3).

**Table 3.** Total number of records and species for each county.

County	Total records	Species richness	County	Total records	Species richness
Kings	28	22	Napa	492	47
Sutter	33	17	Alameda	496	47
San Benito	56	25	San Mateo	504	45
Alpine	93	30	Shasta	514	78
Amador	109	41	Sacramento	524	46
Glenn	111	33	Plumas	530	55
Tehama	123	46	Placer	533	65
Lake	153	48	Fresno	547	54
San Joaquin	157	31	Imperial	562	39
Madera	169	41	Modoc	580	64
San Francisco	177	23	Mono	598	46
Calaveras	179	39	Butte	664	56
San Luis Obispo	180	37	Lassen	668	68
Santa Cruz	191	45	Santa Barbara	701	44
Merced	199	21	Yolo	710	44
Mariposa	209	39	Humboldt	731	57
Del Norte	211	41	Colusa	776	53
Solano	235	38	Nevada	777	56
Sierra	268	48	Mendocino	892	54
Yuba	283	40	Stanislaus	904	42
Trinity	306	50	El Dorado	924	57
Marin	314	40	Sonoma	956	58
Monterey	332	48	San Bernardino	1038	57
Tulare	372	46	Siskiyou	1136	68
Tuolumne	372	45	Santa Clara	1202	51
Orange	437	35	Inyo	1548	59
Contra Costa	445	39	San Diego	1759	58
Ventura	474	35	Los Angeles	1804	45
Kern	487	49	Riverside	2108	58

Most counties supported 40–60 species. Counties that were well above or below the confidence interval may be either relatively species-rich or species-poor (Fig. 3). Siskiyou, Shasta, Inyo, Placer, and Lake Counties were relatively rich in species, while some species-poor counties included Los Angeles, Stanislaus, Yolo, Kern, Colusa, and Ventura (Fig. 3).

**Figure 4.**
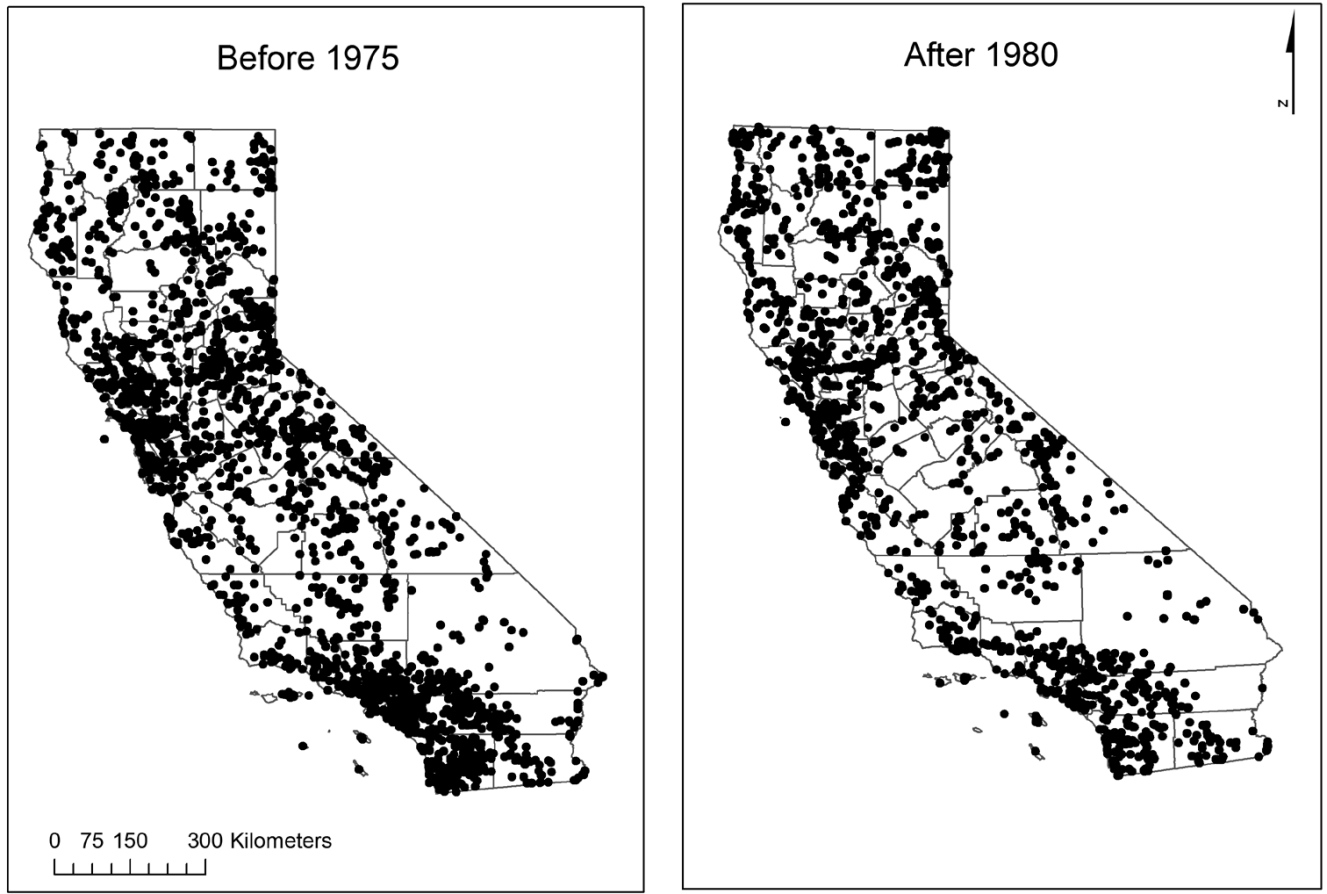
Spatial distribution of California records before 1976, and after 1979.

A map of specimen localities for both time periods demonstrates some additional spatial bias and data gaps (Fig. 4). Dense clusters of records exist around urban centers, including the San Francisco Bay area, Sacramento, and major cities in southern California, such as Santa Barbara, Los Angeles, San Diego, and Riverside. The least sampled and/or occupied area is the desert region in the southeast of the state. While the number of total records was higher before 1976, the spatial distribution of records before 1976 and after 1979 is similar.

### Contribution of collection types to county records

Calbug institutions contributed the highest number of total records with 14,207 total records, followed by observation-based records with 8,269 total records (Table 1). Non-Calbug institutions and private collections provided 5,803 and 3,746 total records, respectively.

**Figure 5.**
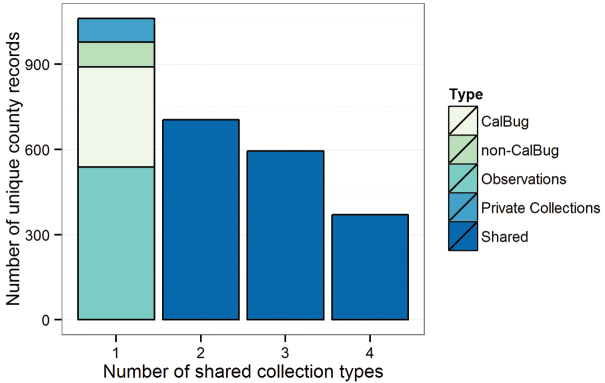
Number of unique county records for each collection type (Calbug collaborating institutions, non-Calbug institutions, observations - Cal Odes and Odonata Central, and private collections), and number of unique county records with two, three, and four shared data types.

The observation-based records contributed the highest number of unique county records with 538 (by species and county only), followed by the Calbug institutions with 353 unique records (Fig. 5). Non-Calbug institutions and private collections contributed 87 and 83 unique county records, respectively. There were 705 county records originated from two of the four collection types, 594 records originated from three types, and 370 records originating from all four collection types (Fig. 5).

### Species occurrence records

There were 8,642 unique species occurrence records (i.e. unique locality and date) before 1976, and 9,175 unique occurrence records after 1979. The most commonly sampled species before 1976 were *Argia vivida*, *Sympetrum corruptum* Hagen, *Libellula saturata*, *Enallagma carunculatum* Morse, and *Ischnura cervula* Selys. The most commonly sampled or observed species after 1979 were *Argia vivida*, *Sympetrum corruptum*, *Ischnura cervula*, *Libellula saturata*, and *Anax junius* (Drury) (Table 4). The least sampled species after 1979 were *Enallagma basidens* Calvert, *Somatochlora albicincta* (Burmeister), *Epitheca spinigera* (Selys), *Stylurus intricatus* (Selys), and *Ophiogomphus severus* Hagen (Table 4). *Aeshna canadensis* Walker, *Tramea calverti* Muttkowski, and *Sympetrum vicinum* (Hagen) were not observed before 1998, 1988, and 1980, respectively. *Enallagma basidens*, *Sympetrum albicincta*, and *Nehalennia irene* (Hagen) were only observed one time prior to 1976 (Table 4).

Thirty-seven species decreased in relative occurrence in the two time periods examined, while 66 species increased (Table 4). Species with the highest increases in relative occurrence were *Anax junius*, *Tramea lacerata* Hagen, *Libellula forensis* Hagen, and *Libellula luctuosa* Burmeister. Species with the greatest declines in relative occurrence were *Argia vivida*, *Sympetrum corruptum*, *Enallagma annexum* (Hagen), *Ischnura denticollis* (Burmeister), and *Enallagma carunculatum* (Table 4). Many of the species with the highest declines are likely the result of differences in sampling approaches in the recent data, much of which were observation-based, as compared to the older specimen data, which was entirely collection-based. Species with the highest declines, that also match patterns of decline in a recent resurvey study by Ball-Damerow et al. (2014), include *Hetaerina americana*, *Sympetrum illotum* (Hagen), *Octogomphus specularis* (Hagen), and *Cordulegaster dorsalis* Hagen.

**Table 4.** Summary of species records, including earliest and latest observation or specimen collection date, unique occurrences (by site and year) before 1976 and after 1979, and the change in relative occurrence in unique records. Bolded records show the same relationship (i.e. increase or decrease in species prevalence) reported in Ball-Damerow et al. (2014). Records that are likely to be a result of taxonomic biases, such as failure to collect common species or spcies that are difficult to identify, and a focus on rare or charismatic species, are indicated by *.

Family	Species	Earliest year	Latest year	Before 1975	After 1980	Change
Coenagrionidae	Argia vivida*	1879	2013	767	535	-232
Libellulidae	Sympetrum corruptum*	1892	2013	612	414	-198
Coenagrionidae	Enallagma annexum*	1900	2013	268	134	-134
Coenagrionidae	Ischnura denticollis*	1900	2013	256	126	-130
Coenagrionidae	Enallagma carunculatum*	1900	2013	329	218	-111
Coenagrionidae	*Amphiagrion abbreviatum*	1904	2013	168	70	-98
**Calopterygidae**	***Hetaerina americana***	**1879**	**2013**	**304**	**220**	-**84**
Coenagrionidae	Argia nahuana*	1894	2013	115	35	-80
**Libellulidae**	***Sympetrum illotum***	**1892**	**2013**	**270**	**205**	**-65**
Coenagrionidae	Enallagma praevarum*	1900	2013	103	67	-36
**Gomphidae**	***Octogomphus specularis***	**1900**	**2013**	**97**	**61**	**-36**
Coenagrionidae	Enallagma civile*	1926	2013	195	167	-28
Libellulidae	Pantala hymenaea*	1912	2013	141	114	-27
**Cordulegastridae**	***Cordulegaster dorsalis***	**1900**	**2013**	**139**	**118**	**-21**
Coenagrionidae	*Telebasis salva*	1900	2013	86	63	-23
Coenagrionidae	Enallagma boreale*	1903	2013	92	71	-21
Libellulidae	Paltothemis lineatipes*	1914	2013	103	84	-19
**Lestidae**	***Archilestes californicus***	**1900**	**2012**	**61**	**48**	**-13**
**Libellulidae**	***Libellula nodisticta***	**1894**	**2013**	**51**	**39**	**-12**
Libellulidae	*Libellula comanche*	1914	2013	50	38	-12
**Lestidae**	***Lestes congener***	**1900**	**2013**	**64**	**53**	**-11**
**Lestidae**	***Lestes dryas***	**1910**	**2013**	**89**	**80**	**-9**
**Libellulidae**	***Sympetrum pallipes***	**1894**	**2013**	**130**	**125**	**-5**
Libellulidae	*Leucorrhinia hudsonica*	1914	2013	42	32	-10
Coenagrionidae	Enallagma anna*	1915	2012	26	19	-7
Coenagrionidae	Enallagma clausum*	1938	2013	19	12	-7
Libellulidae	*Plathemis subornata*	1915	2013	34	28	-6
**Libellulidae**	***Sympetrum danae***	**1914**	**2013**	**33**	**27**	**-6**
Coenagrionidae	*Ischnura barberi*	1897	2013	59	55	-4
**Gomphidae**	***Ophiogomphus bison***	**1907**	**2013**	**58**	**55**	**-3**
**Libellulidae**	***Sympetrum obtrusum***	**1914**	**2013**	**39**	**36**	**-3**
Libellulidae	*Libellula croceipennis*	1914	2013	22	19	-3
Aeshnidae	*Aeshna walkeri*	1900	2013	41	40	-1
Lestidae	*Archilestes grandis*	1897	2012	25	24	-1
Libellulidae	Erythemis collocata*	1900	2013	216	227	11
**Libellulidae**	***Sympetrum semicinctum***	**1909**	**2013**	**61**	**63**	**2**
**Coenagrionidae**	***Coenagrion resolutum***	**1914**	**2011**	**13**	**13**	**0**
Aeshnidae	*Aeshna interrupta*	1914	2013	50	53	3
Lestidae	*Lestes disjunctus*	1912	2013	62	66	4
Coenagrionidae	Ischnura gemina*	1900	2013	12	13	1
Gomphidae	*Stylurus intricatus*	1915	2012	6	7	1
Gomphidae	*Erpetogomphus compositus*	1914	2013	48	52	4
Lestidae	*Lestes unguiculatus*	1914	2013	10	13	3
Coenagrionidae	*Enallagma basidens*	1974	2012	1	4	3
Corduliidae	*Cordulia shurtleffii*	1914	2013	32	37	5
Coenagrionidae	*Argia hinei*	1915	2013	12	16	4
Gomphidae	Stylurus plagiatus*	1965	2013	4	8	4
Corduliidae	*Epitheca spinigera*	1914	2013	2	6	4
Corduliidae	*Somatochlora albicincta*	1952	2013	1	5	4
Coenagrionidae	*Argia moesta*	1938	2013	17	22	5
Libellulidae	*Orthemis ferruginea*	1935	2013	16	21	5
Gomphidae	Ophiogomphus severus*	1914	2013	3	8	5
**Gomphidae**	***Progomphus borealis***	**1900**	**2013**	**61**	**70**	**9**
Libellulidae	Sympetrum internum*	1914	2013	12	18	6
Coenagrionidae	*Argia alberta*	1915	2013	19	26	7
Coenagrionidae	Nehalennia irene*	1973	2013	1	9	8
**Lestidae**	***Lestes stultus***	**1903**	**2013**	**45**	**56**	**11**
Gomphidae	*Erpetogomphus lampropeltis*	1915	2013	10	19	9
Gomphidae	Ophiogomphus morrisoni*	1914	2013	23	33	10
**Libellulidae**	***Libellula saturata***	**1879**	**2013**	**354**	**385**	**31**
Libellulidae	Sympetrum madidum*	1897	2013	59	72	13
Corduliidae	*Somatochlora semicircularis*	1914	2013	21	32	11
Libellulidae	*Libellula quadrimaculata*	1914	2013	80	95	15
Coenagrionidae	*Argia sedula*	1945	2013	26	38	12
Coenagrionidae	*Zoniagrion exclamationis*	1911	2013	51	65	14
Libellulidae	Libellula composita*	1915	2013	11	23	12
Aeshnidae	*Aeshna canadensis*	1998	2012	0	12	12
Coenagrionidae	*Ischnura erratica*	1900	2013	15	29	14
Coenagrionidae	*Ischnura hastata*	1938	2013	4	18	14
Libellulidae	*Tramea calverti*	1988	2011	0	14	14
Gomphidae	Stylurus olivaceus*	1914	2012	5	21	16
Libellulidae	*Macrodiplax balteata*	1947	2013	2	19	17
Libellulidae	Leucorrhinia glacialis*	1914	2013	15	33	18
Libellulidae	Sympetrum costiferum*	1934	2013	11	29	18
Aeshnidae	Aeshna palmata*	1914	2013	34	54	20
Gomphidae	Ophiogomphus occidentis*	1914	2013	17	36	19
Libellulidae	*Sympetrum vicinum*	1980	2012	0	19	19
Calopterygidae	*Calopteryx aequabilis*	1951	2013	7	27	20
Libellulidae	*Brachymesia furcata*	1930	2013	7	28	21
Libellulidae	*Ladona julia*	1953	2013	4	25	21
**Libellulidae**	***Pachydiplax longipennis***	**1900**	**2013**	**189**	**222**	**33**
Aeshnidae	*Aeshna umbrosa*	1915	2012	16	40	24
Coenagrionidae	*Ischnura ramburii*	1930	2013	7	32	25
Libellulidae	*Leucorrhinia intacta*	1918	2013	15	44	29
Coenagrionidae	*Argia agrioides*	1907	2013	71	104	33
Libellulidae	*Perithemis intensa*	1934	2013	8	38	30
**Coenagrionidae**	***Ischnura perparva***	**1898**	**2013**	**247**	**292**	**45**
Gomphidae	*Gomphus kurilis*	1905	2013	68	104	36
Corduliidae	Macromia magnifica*	1900	2013	27	61	34
Libellulidae	*Pantala flavescens*	1915	2013	20	55	35
Coenagrionidae	*Argia lugens*	1901	2013	86	126	40
Aeshnidae	Anax walsinghami*	1915	2013	19	56	37
Libellulidae	*Brechmorhoga mendax*	1901	2013	31	69	38
Libellulidae	*Tramea onusta*	1907	2013	31	69	38
Petaluridae	Tanypteryx hageni*	1918	2013	22	61	39
Libellulidae	*Plathemis lydia*	1912	2013	157	208	51
**Coenagrionidae**	***Argia emma***	**1910**	**2013**	**72**	**119**	**47**
Aeshnidae	*Rhionaeschna californica*	1897	2013	92	144	52
**Coenagrionidae**	***Ischnura cervula***	**1902**	**2013**	**317**	**394**	**77**
Corduliidae	*Epitheca canis*	1914	2013	16	77	61
**Aeshnidae**	***Rhionaeschna multicolor***	**1898**	**2013**	**257**	**345**	**88**
Libellulidae	*Libellula pulchella*	1905	2013	84	166	82
**Libellulidae**	***Libellula luctuosa***	**1929**	**2013**	**54**	**143**	**89**
Libellulidae	*Libellula forensis*	1900	2013	85	220	135
**Libellulidae**	***Tramea lacerata***	**1900**	**2013**	**107**	**254**	**147**
**Aeshnidae**	***Anax junius***	**1900**	**2013**	**196**	**361**	**165**
Total number of unique occurrences:				8642	9175	

In comparing the average and range of latitude and elevation across individual species occurrence localities, we excluded all records with an error radius of greater than 4 km. The total number of unique records before 1976 available was then 5,142 and the total number of unique records after 1979 was 7,785. The median average latitude across all species increased by 0.7° (±0.82, p<0.001), indicating an average shift of around 78 km northwards (Table 5). Average minimum latitude declined slightly by 0.12° (±1.1, p=0.01), and average maximum latitude increased by 0.59° (±1.3, p<0.001, Table 5). Neither average nor average maximum elevation across species changed significantly over the two time periods, but average minimum elevation declined by 108 m (±360 m, p=0.003; Table 5).

**Table 5.** Summaries of change in unique species latitude and elevation values before 1976 and after 1979. Unique records represent unique combinations of species, locality coordinates, and year. Records included in this assessment have an error radius ≤ 4 km.

	Average change	Standard deviation	Wilcoxon rank-sign test	P-Value
**Avg Latitude**	**0.70° (78 km)**	**0.82**	**V = 542**	**<0.001**
**Min Latitude**	**-0.12° (-13 km)**	**1.12**	**V = 3429**	**0.01**
**Max Latitude**	**0.59° (65 km)**	**1.28**	**V = 643**	**<0.001**
Avg Elevation (m)	-49	248	V = 2730	0.37
**Min Elevation (m)**	**-108**	**360**	**V = 3327**	**0.003**
Max Elevation (m)	49	613	V = 2099	0.19

## Discussion

The California Odonata database provides an overview of common patterns to be expected in the temporal distribution of museum records in California. For odonates, peaks in specimen acquisition occurred in 1914–15 as a result of C.H. Kennedy’s work (Kennedy 1917), with subsequent peaks in the 1950s, 1960s and 1970s through the combined work of several collectors. After this mid-20^th^ century time period, specimen acquisition was slower. The largest peak in the Odonata database has occurred since 2000, and represents mostly observation-based records obtained from odonate enthusiasts.

Previous work has noted a decline in specimen acquisition of natural history museums over the past 30–40 years that corresponds with declines in funding for many of these institutions (Pyke and Ehrlich 2010). However, observation-based records now provide a valuable complement to specimen records in documenting change in species prevalence and distribution, especially when such records are photo-vouchered and vetted (e.g. Breed et al. 2013, Pyke and Ehrlich 2010, Soberon et al. 2000).

The present study also identified spatial biases and data gaps, which should be addressed in any distributional analyses and in designing future sampling investigations of California odonates. As demonstrated in a previous spatial analysis of Odonata collection data in North America, collections are often located near more highly populated regions (e.g. Hassall and Thompson 2010). Sampling locations for California odonates are clustered around urban areas, such as the San Francisco Bay area, Sacramento, Los Angeles, and San Diego. The more sparsely populated desert region in the southeast has very few records, which may also be the result of a lack of freshwater habitat in the region (Fig. 4).

Species richness is not strongly associated with total number of records at the statewide scale (Fig. 2), while it is at the county scale (Fig. 3). During the 1980s and 1990s, there was a significant drop in the total number of records without a parallel drop in species richness. It seems that after 1,500 records species richness for the state levels off at around 100 species, which is close to the total number known resident species in the state (106 species). Even in 1980, with 1,265 total records, species richness dropped only to 77 species (Fig. 2). There is a stronger exponential relationship between the total number of records and species richness observed in a given county (Fig. 3). While species richness leveled off at around 58 species per county with at least 600 records, there were some obvious outliers that could represent relatively species rich or poor counties. In particular, Shasta County had 78 species recorded with only 514 records, which is likely because it is located in the warmest region with relatively high precipitation and aquatic habitat. In contrast, counties with below average species richness given the number of records were all dry regions in the Central Valley or southern California. Similarly, Hassall and Thompson (2010) found that collection effort, in addition to warm temperature and water availability, plays a major role in species richness of odonates observed in various regions of North America. Future sampling, particularly in under-sampled regions and in warm areas with higher freshwater habitat availability (e.g. Sutter County and Lake County), is therefore likely to yield additional species.

Each of the different collection types—Calbug (i.e. California) institutions, non-Calbug institutions, private collections, and observation-based records—contributed significantly to the total number of records and to county records for species. The Calbug institutions had the highest total number of records, followed by observation-based records, which had just over half the number of total records as Calbug. However, observations contributed significantly more county records for species. The goal of many enthusiasts is to find new county records, which likely explains this difference. We find that recent observation-based records have greatly contributed to our knowledge of the spatial distribution of odonate species in California.

Apparent changes in species prevalence according to occurrence records are sometimes the result of variation in taxonomic biases, particularly in comparing natural history specimens and observation-based records (Table 4). According to existing occurrence records, two species with the highest decline in prevalence over time were two of the most common species in the state, *Argia vivida* and *Sympetrum corruptum*. Many individuals reporting species observations to CalOdes or Odonata Central may have neglected these species in at least some of their lists, perhaps because these collectors considered less-common species to be more interesting or noteworthy. Another potential problem with observation-based data is the difficulty in identifying certain species in the field. In general, the most difficult group to identify is the genus *Enallagma* (particularly *Enallagma boreale* and *Enallagma annexum*), and many enthusiasts report them as *Enallagma* sp. or as “bluets”. Less experienced enthusiasts in particular may avoid reporting this group or other difficult to identify species, such as *Argia agrioides* and *Argia nahuana*. In contrast, Odonata taxonomists contributing to specimen records from the early and mid-20^th^ century often focused on these groups, which were in need of taxonomic revision (e.g. Garrison 1984). As a result of this known discrepancy, such species should not be included in comparing specimen and observation-based data unless analysis methods address collecting biases, or only include results of certain collectors less likely to demonstrate this taxonomic bias. In general, charismatic, rare, and colorful species are often more likely to be present in both specimen collections and in observation-based lists (e.g. Dunn 2005).

Species that have increased in prevalence over time, however, often demonstrate more reliable results than those with apparent declines (Szabo et al. 2010). Many of the species with the highest increases in relative occurrence also demonstrated increased prevalence in a recent resurvey study (Ball-Damerow et al. 2014, Table 4). Eight out of the ten species with the highest increases in prevalence were habitat generalists, nine species were widespread throughout the state, and all ten were found across a wide range of elevation from sea level to around 2,000 m. Similarly, previous studies have demonstrated that widespread, habitat generalist species have expanded considerably over time (Ball-Damerow et al. 2014, Dupont et al. 2011, Julliard et al. 2004, Korkeamaki and Suhonen 2002). The two most conspicuous migratory species, *Anax junius* and *Tramea lacerata*, demonstrated the highest increases in prevalence. In a related resurvey study, Ball-Damerow et al. (2014) found that four out of the five migratory species in the state were among those with the highest increases in prevalence, including *Anax junius* and *Tramea lacerata*. The other two migratory species that increased in the resurvey study were *Sympetrum corruptum* and *Pantala hymenaea*, both of which are more drab-colored, less conspicuous, and may therefore be less reported in recent observation-based lists (Ball-Damerow et al. 2014).

Odonata species in California have expanded northwards by an average of around 78 km and demonstrated an average increase in northern range margins of 65 km. This shift is unlikely to be the result of location bias, considering that overall distribution of sampled sites was similar across the two time periods (Fig. 4), and favorite collecting sites are not likely to shift north in this way. Similarly, a study of 37 species of British Odonata showed a northward shift at the range margin of about 74 km when comparing records from 1960–70 and 1985–1995 (Hickling et al. 2005). Overall, a wide range of taxa are shifting northwards and to higher elevations as a result of increasing temperatures (e.g. Angert et al. 2011, Hickling et al. 2006, Parmesan 2006).

However, we also observed a decline in the average minimum elevation across species. This could be the result of increases in dry-season water habitats throughout low elevation areas of the Central Valley with increased irrigation for agriculture (Ball-Damerow et al. 2014). This region of the state was previously drier and may have supported fewer odonates in the early 20^th^ century. In contrast, mountainous regions generally have higher rainfall and more natural aquatic habitat. The unexpected decline in elevation could also be a result of more recent spatial bias to collect near centers of human population, which also tend to occur at lower elevations.

## Conclusions

The California Odonata database is one of the largest state-level databases for this order of insects in North America. This database provides a valuable source of information to determine change in Odonata communities and species distribution in the region over time. The timespan of the collection, from the late 1800s through 2013, coincides with unprecedented human population growth, redistribution of water throughout an agriculture-intensive state, and large-scale land use change (Mount 1995). One of the most powerful applications of this database is its use as a data-exploration tool. For example, researchers may identify particular species, regions, or even collectors that warrant further study or that may be amenable to analyses of change over time. Further investigation will undoubtedly yield discoveries concerning changes in Odonata biology and distribution over time. Moreover, comparisons of our California odonate data to that of other regions or groups of organisms may provide insight into the general use of Odonata as biological indicators of change over time and more general principles of global change biology.

## Supplementary materials

Supplementary material 1 California Odonata database records after data processing, as described in methods. Data type: occurence This dataset is made available under the Open Database License (http://opendatacommons.org/licenses/odbl/1.0/). The Open Database License (ODbL) is a license agreement intended to allow users to freely share, modify, and use this Dataset while maintaining this same freedom for others, provided that the original source and author(s) are credited.Joan E. Ball-Damerow, Peter T. Oboyski, Vincent H. Resh
